# Formulation of Quercetin Mouthwash and Anti-microbial Potential Against Critical Pathogens: An In-Vitro Evaluation

**DOI:** 10.7759/cureus.51688

**Published:** 2024-01-05

**Authors:** Akshayaa L, Jishnu Krishna Kumar, Rajeshkumar Shanmugam

**Affiliations:** 1 Public Health Dentistry, Saveetha Dental College and Hospitals, Saveetha Institute of Medical and Technical Sciences, Saveetha University, Chennai, IND; 2 Community Dentistry, Saveetha Dental College and Hospitals, Saveetha Institute of Medical and Technical Sciences, Saveetha University, Chennai, IND; 3 Pharmacology, Saveetha Dental College and Hospitals, Saveetha Institute of Medical and Technical Sciences, Saveetha University, Chennai, IND

**Keywords:** oral diseases, fostering innovation, affordable medicines, pathogens, antimicrobial, quercetin, mouthwash

## Abstract

Introduction

Herbal mouthwashes were designed and prepared using essential oils from phytotherapeutic plants, containing active agents such as catechins, tannins, and sterols. Quercetin is one of the most abundant natural flavonoids predominantly found in foods including fruits, and vegetables. It has wide biological effects such as antioxidant, anti-aging, anti-inflammatory, probiotic, and metabolic modulation activities. Research has pointed toward its anti-microbial efficacy and bio-compatibility.

Materials and methods

A sample of commercially available Himalaya Hiora mouthwash (control) and 0.02 mg of Quercetin (test sample) that is sold commercially were procured. Next, 0.01 grams of sodium lauryl sulfate (SLS), which serves as the foaming agent, 0.001 grams of preservative (sodium methylparaben), and 0.3 grams of sucrose, the sweetening agent, were added to the test sample to formulate the mouthwash. We added 0.5 milliliters of the prepared Quercetin solution to the mixture. The effectiveness of the Quercetin mouthwash formulation as an antimicrobial was evaluated using the Agar-Well Diffusion Method against five oral pathogens and compared to the Hiora mouthwash.

Results

On quantifying the zone of inhibition, it was observed that at 100 μL of concentration, *Staphylococcus aureus* shows maximum inhibition rate, i.e., 15 mm when compared to commercially available herbal mouthwash (Himalaya Hiora). Thus, from the results obtained, we found that when concentration increases there is a significant zone of inhibition shown by oral pathogens.

Conclusion

Quercetin mouthwash formulation has proven to have a good antimicrobial effect when compared to standard mouthwash. The effective antimicrobial activity suggests its potential use as an adjuvant chemical plaque control modality. Further clinical trials would pave the way for its use as a routine or therapeutic antimicrobial agent.

## Introduction

Herbal mouthwashes were designed and prepared using essential oils from phytotherapeutic plants, containing active agents such as catechins, tannins, and sterols. One of the naturally occurring plant flavonoids, quercetin is present in a wide variety of foods, including fruits, vegetables, tea, and wine [1]. Flavonoids are secondary metabolites that have been described as health-promoting, disease-preventing dietary supplements that act as cancer-preventive agents [2,3]. The most common vegetable used both as an edible food and a therapeutic herb is the onion (Allium cepa), which contains the highest concentration of quercetin. Quercetin is found in several therapeutic plants, including Hypericum perforatum and Ginkgo biloba. The chemical compound's penta-hydroxyflavone, which has various biologically advantageous properties including antioxidant, anti-aging, anti-inflammatory, prebiotic, and metabolomic modulating activities, makes up the majority of the chemical structure. Numerous pharmacological effects of quercetin include antioxidant, anti-cancer, antiviral, antibacterial, neuroprotective, anti-inflammatory, and cardiovascular. Also, it is recently recognized as “generally recognized as safe” (GRAS) status by the United States Food and Drug Organization [4-6].

Quercetin is a polyphenolic flavonoid generated from plants that have been connected to both human and animal health advantages. We studied the impact of orally administered quercetin on host inflammatory response, oral microbial composition, and periodontal disease phenotype using a systematic approach [7]. In vivo, studies stated that quercetin supplementation has reduced alveolar bone loss, gingival cytokine expression, and inflammatory cell infiltration [8]. Dental caries is a complex dental disease that needs a vulnerable host, causative microorganisms, and an appropriate substrate. Plaque is the major etiology of dental caries, gingivitis, and periodontal diseases. Two major etiological factors of periodontal disease are specific bacterial species that colonize subgingival sites and cause subsequent host inflammatory and immune responses to the periodontopathogens [8,9]. For an effective plaque control chemical agents are critical. A multimodal orally deliverable mechanism such as essential oils from phytotherapeutic plants, which include active ingredients including catechins, tannins, and sterols could be used to prepare herbal mouthwashes [10]. Bioactive agents like Quercetin might prove beneficial as an adjunct to oral plaque control agents.

*Staphylococcus aureus*, *Escherichia coli*, and *Pseudomonas aeruginosa* are only a few of the Gram-positive and negative bacteria that are resistant to quercetin [11,12]. Despite the existence of numerous preventative methods, such as fluoride toothpaste, dental sealants, and mouthwashes, dental caries remains widespread and need to be prevented. The essential oils from phytotherapeutic plants, which include active ingredients including catechins, tannins, and sterols were used to prepare herbal mouthwashes. Mouthwashes have the power to deliver medicinal components and can be utilized to access organisms that are present on the mouth's surface [13]. Numerous researchers have investigated quercetin's antibacterial properties in depth and have looked into it as a potential treatment for a variety of oral pathogens. Recent investigations have shown that quercetin can disrupt the integrity of the bacterial cell membrane, thereby inhibiting bacterial growth [14,15]. Additionally, the cariogenic strains of *Lactobacillus acidophilus* (*L. acidophilus*) and *Streptococcus mutans* (*S. mutans*) prevented from proliferating the vesicles made with the highest concentration of mint oil. Another study done by Wang et al. reported that Quercetin compounds destroy the structural integrity of the cell wall and membrane of *S. aureus* and *E. coli* [16,17]. We studied the impact of formulated Quercetin mouthwash on oral microbial composition (antimicrobial activity), and thus, dental caries and periodontal disease. The purpose of the current study was to formulate a Quercetin-based mouthwash and to examine its antimicrobial effect against critical oral pathogens.

## Materials and methods

Ethics approval

The protocol was reviewed and approved by the Ethical and Research Committee at Saveetha Dental College and Hospitals. The formally constituted Institutional Review Board approved the study with approval number EC/NEW/INST/2021/1967, as it did not involve any human subjects and followed the ethical principles of the Declaration of Helsinki updated in 2013. The study was in-vitro in nature and did not involve any foreseeable risks.

Sample details

Quercetin compound (USP1592409) had been procured from the Sigma-Aldrich ©Merck KGaA. The study received ethical clearance from the Institutional Review Board (IRB) as no human subjects were involved in the study. Hence, IRB approval number EC/NEW/INST/2023/1967 was assigned. The microorganisms' standard strains were obtained from the microbiological depository and were denoted as per Microbial Type Culture Collection and Gene Bank (MTCC) number. This study used the following strains of microorganisms: *Candida albicans* (Ca), MTCC 2091; *Enterococcus faecalis* (Ef), MTCC 35550; *S. aureus* (Sa); *S. mutans*, MTCC 12598; and *Lactobacillus*, MTCC 10307.

Sample testing material

Commercially procured Quercetin compound (USP1592409) Sigma-Aldrich©Merck KGaA, 0.02mg has been separated by weighing in a weighing machine and 10 mL of ethanol (≥99% purity) has been pipetted and taken separately.

Mouthwash preparation

A sample of 0.02 mg of commercially available Quercetin was mixed with 10 ml of ethanol. Following that, 0.01 grams of sodium lauryl sulfate (SLS), which serves as the foaming agent, 0.001 grams of preservative (sodium methylparaben), 0.3 grams of sucrose, and the sweetening agent, were added. To the prepared solution, 0.5 mL of prepared Quercetin solution was added. Further antimicrobial activity of mouthwash was evaluated.

Antimicrobial activity of Quercetin mouthwash

The Quercetin mouthwash's antimicrobial efficacy was evaluated using the Agar-Well Diffusion Method. Mouthwash dilutions were tested against five different organisms: *C. albicans*, *S. aureus*, *E. faecalis*, *S. mutans*, and *Lactobacillus*. Their newly made Quercetin suspensions were spread out on the Muller-Hinton Agar plates (which contain the bacteria *S. mutans*, *S. aureus*, *E. faecalis*, and *Lactobacillus*) and the Rose Bengal Agar plate (which contains the bacteria *C. albicans*). After adding the prepared Quercetin concentrations of 25, 50, and 100 μL to the wells, the plates were incubated for 24 hours at 37°C in 5% CO_2_ (v/v) for 24 h. After the plates were incubated, the minimum inhibitory concentration zone surrounding each well was measured. Following the use of herbal mouthwash that is readily available in stores as a positive control, zones of inhibition were noted for every well. Zones of inhibition were then noted for every well where commercially available herbal Himalaya Hiora mouthwash Manufactured by the Himalaya Drug Company Makali, Bangalore, India, was used as a positive control. The control was chosen considering its natural ingredients and its comparable efficiency to Chlorhexidine [18]. Each gram of HiOra mouthwash contains Salvadora persica - 5.0 mg, Piper betel - 10 mg, Terminalia bellerica - 10 mg, Gandhapura taila - 1.2 mg, Ela - 0.2 mg, Peppermint satva - 1.6 mg, and Yavanisatva - 0.4 mg.

Statistical analysis

The collected data were entered into Microsoft Excel 2017, and IBM SPSS Statistical software for Windows, version 21 (IBM Corp., Armonk, NY, USA) was used for analysis. For continuous variables, the descriptive statistics were shown as mean ± standard deviation. The Shapiro-Wilk test was used for normality testing, and the independent sample t-test was used for univariate analysis to compare the variations in quercetin mouthwash's antibacterial activity. Tables and graphs were created from the tabulated results. A p-value of less than 0.05 was deemed statistically significant.

## Results

**Figure 1:**
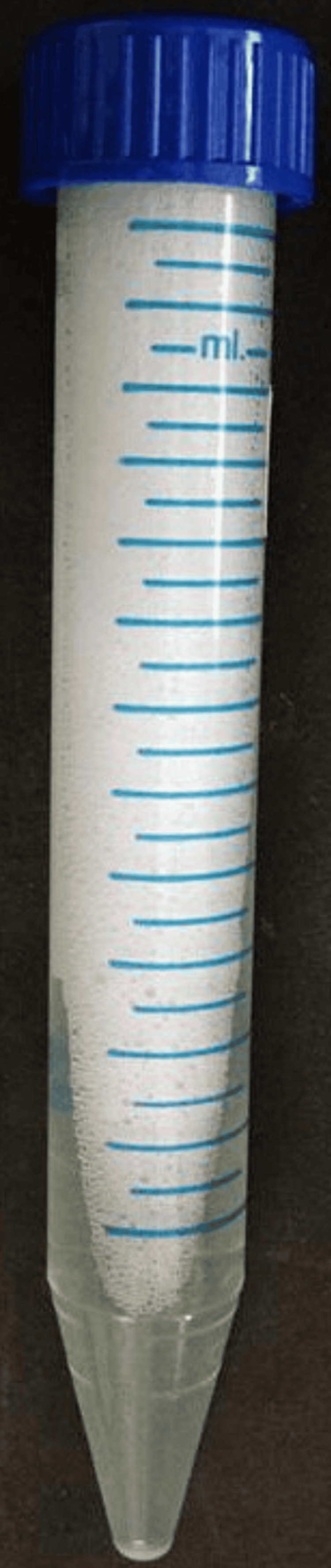
Formulated Quercetin-based mouthwash

Figure 1 presents the formulated Quercetin mouthwash which would be utilized to develop various test concentrations of 25, 50, and 100 microliters. These concentrations of mouthwash were tested against five potential oral pathogens with commercial mouthwash as the control.

**Figure 2:**
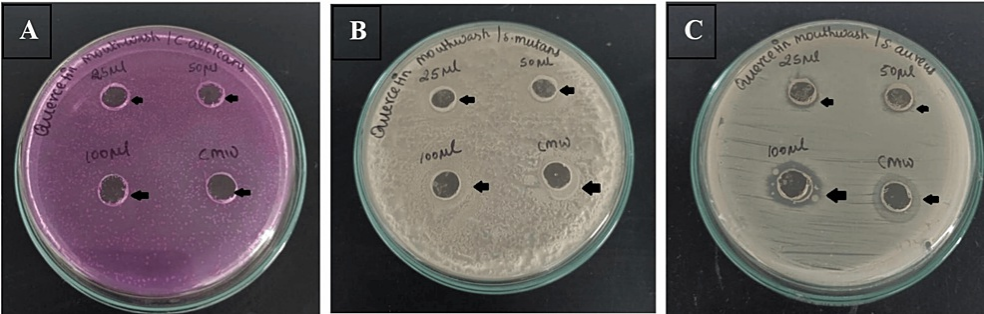
Antimicrobial activity of various concentrations of Quercetin mouthwash and control Inhibition zone (mm) on culture plates presented by Quercetin mouthwash at 25, 50, 100 μL and control on Candida albicans (A), antimicrobial activity on Streptococcus mutans (B), and on Staphylococcus aureus (C).

Figures 2A-2C depict the Agar well diffusion plates, and its zone of inhibition; the antimicrobial activity of the formulated Quercetin mouthwash against *S. mutans*, *S. aureus*, and *C. albicans* can be seen after 24 hours of incubation.

**Figure 3:**
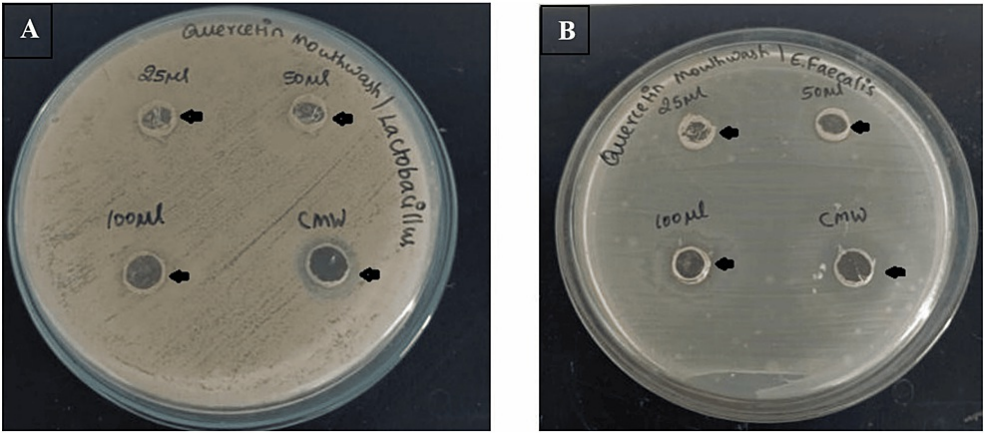
Inhibition halo noticed on Lactobacillus and Enterococcus faecalis Distinction of the inhibition halo diameter after 24 hours, in the presence of 25, 50, and 100 μL of formulated Quercetin mouthwash, against Lactobacillus (A) and Enterococcus faecalis (B). Arrow indicates the zone of inhibition.

Figures 3A, 3B represent the antimicrobial effect of Quercetin mouthwash against *Lactobacillus* and *E. faecalis* incubated on agar well diffusion plates after 24 hours of incubation. A zone of inhibition can be noticed as a Halo on the plates.

Table 1 presents the comparative antimicrobial effect of Quercetin against *C. albicans*, *S. mutans*, *Lactobacillus*, *S. aureus*, and *E. faecalis*. It is observed that at 100 μL of concentration, *S. aureus* shows maximum inhibition rate, i.e., 15 mm, compared to standard mouthwash. Also, it can be found that when concentration increases there is a significant zone of inhibition shown on the culture plates.

**Table 1:** Descriptives of Zone of Inhibition of Test and Control group Objective representation of Zone of Inhibition among test group (Quercetin mouthwash) at 25μL, 50μL, 100μL and control group (commercial mouthwash) as evaluated on culture plates.

S. No.	Tested microbes	25 μL	50 μL	100 μL	Control
1	C. albicans	09 mm	09 mm	09 mm	12 mm
2	S. mutans	09 mm	09 mm	09 mm	09 mm
3	S. aureus	10 mm	12 mm	15 mm	13 mm
4	Lactobacillus	09 mm	09 mm	09 mm	12 mm
5	E. faecalis	09 mm	09 mm	09 mm	12 mm

Table 2 depicts the comparative mean analysis between the test and control groups of prepared Quercetin and commercial mouthwash. In comparison, the p-value was found to be 0.310 (>0.05), which shows a statistically insignificant difference between the two groups. It suggests a potential antibacterial efficacy of the test formulation (Quercetin mouthwash) compared to the control (commercial herbal mouthwash).

**Table 2:** Inferential analysis of minimum inhibitory concentration (μL) among test and positive control groups Comparison of means using Independent sample t-test. The p-value was found to be 0.310 (>0.05) which infers to be statistically not significant. Test group composed of Quercetin-based mouthwash and positive control composed of conventional mouthwash.

Comparative analysis of minimum inhibitory concentration (μL)				
Control Group (Commercial mouthwash)		Test Group (Quercetin mouthwash)		p-value
Mean	Standard deviation	Mean	Standard deviation	0.310*
10.20	2.67	11.60	1.51	

## Discussion

An earlier study conducted by Murugan et al. assessed the antimicrobial effects of pure Quercetin which was isolated from a suspension culture of *C. pulcherrima* against several bacterial and fungal infections [19]. According to our study, Quercetin showed a higher inhibitory effect against *S. aureus* and *E. faecalis*. These findings imply that Quercetin might be employed as a natural antibacterial agent. Future research will focus on tracing the molecular mechanism of Quercetin's antibacterial potentiality against the tested pathogens [20]. In our study, Quercetin was found to have good antibacterial activity against *C. albicans*, *S. mutans*, *Lactobacillus*, *S. aureus*, and *E. faecalis* at a concentration of 100 μL. Numerous investigations have shown that the preparation of Quercetin vesicles combined with the maximum amount of mint oil prevented the growth of cariogenic microbes [21].

Another study stated that Quercetin supplements promote a healthy oral microenvironment and balanced periodontal tissue homeostasis by reducing inflammation and promoting the growth of symbiotic bacteria. In agreement herbal mouthwashes have shown a comparable reduction in microbial colony count when compared to Chlorhexidine mouthwash [18]. Further, the study showed the significant effect of herbal mouthwash such as *Hiora* in reducing plaque micro-organisms and thus gingivitis. This concept in the study offers crucial support for translational research to enhance oral health [22,23]. A study done by Pandya et al. reported the use of topically applied Quercetin gel has produced positive outcomes when compared to benzydamine hydrochloride in patients with minor recurrent aphthous and further research with this drug entity might prove beneficial [24].

Many in-vivo studies have stated that Quercetin has lowered the severity of lesions and inflammation and also prevented the side effects of chemotherapy-induced oral mucositis using 5-fluorouracil [21,25]. According to a study by Hossion et al., Quercetin glycosides which have been artificially created and synthesized proved to inhibit the growth of *P. aeruginosa*, *S. aureus*, and *E. coli* species [26]. Recent studies have revealed that the main antibacterial mechanisms of Quercetin include destroying the bacterial cell walls, altering cell permeability, impacting protein synthesis, lowering enzyme activity, and inhibiting nucleic acid synthesis. [27,28]. Only a few studies have reported that Quercetin can be used as a promising compound in the preparation of mouthwash [29]. Future research could focus on tracing the molecular mechanism of Quercetin's antibacterial potentiality against the tested pathogens. Thus, the future scope of our research is to include large-scale clinical trials that can prove the feasibility of Quercetin-based mouthwash as an alternative to commercially available mouthwash.

The inability to formulate mouthwashes with varying concentrations of Quercetin due to budgetary restrictions was one of the study's limitations, as to avoid any potential conflict no external funding was considered. The invitro analysis provided insight into the comparable antimicrobial efficacy of the test formulation and its suitability for large-scale pre-clinical evaluation. The results may be helpful in future research examining clinical perspectives of the potential use of Quercetin as a plaque control agent.

## Conclusions

The present study elaborated on the antimicrobial efficacy of the potential oral pathogens. Quercetin mouthwash formulation has proven to have a potential broad-spectrum bactericidal activity comparable to commercially available herbal mouthwash. We observed that when the concentration of mouthwash increases, a significant zone of inhibition is created against oral pathogens. Therefore, the current research warrants extensive clinical trials and research on long-term effects.
